# Conducting Co-Design with Older People in a Digital Setting: Methodological Reflections and Recommendations

**DOI:** 10.5334/ijic.6546

**Published:** 2022-12-09

**Authors:** Andrew Darley, Áine Carroll

**Affiliations:** 1 University College Dublin, School of Medicine, Health Sciences Centre, Belfield, Ireland; 2 The National Rehabilitation Hospital, Dublin Ireland

**Keywords:** co-design, patient and public involvement, qualitative methods, ageing, digital health, digital literacy

## Abstract

**Introduction:**

Co-design has been identified as a participatory method to create person-centred integrated healthcare services that align with older people’s values and lived experiences.

**Description:**

Existing guidelines on conducting co-design primarily focus on in-person methods with limited guidance on using digital methods to collect data. This gap in knowledge is particularly pertinent when co-designing with older people who can experience challenges with digital literacy and accessibility. This article uses the exemplar of a pilot site within a European co-design research project, aiming to create digital health technology to support integrated care, to describe the steps and considerations required when collaborating with older people in an online environment. Focus groups and one-to-one interviews were conducted utilising digital mediums of teleconferencing and telephone calls to engage and collaborate with older people.

**Discussion:**

Several preparatory steps are required to effectively bridge the digital divide and conduct co-design with older people including engaging gatekeepers, relationship and trust-building, assessing digital literacy levels, education and providing technological support.

**Conclusion:**

This article highlights the steps and considerations that researchers should be aware of when embarking on co-designing with older people in a digital setting. The authors describe their methods that promotes inclusivity and the empowerment of older people as equal collaborators in the research process. The co-design approach and recommendations can be applied to various research settings and wider areas of integrated care with this population.

## Introduction

An evident shift in healthcare improvement has occurred in recent years due to the recognised value of working in partnership with people to create policies and services which they intend to serve [1]. Patient and public involvement means that the patient experience is an integral component of health and social care planning and creating solutions to address the specific challenges they face [2]. Co-design within a healthcare context is a specific value-driven approach that seeks to educate, empower and iteratively collaborate, both with patients and care providers as equal partners to create a solution that is rooted in their lived experience, rather than assuming what may be beneficial to them [1,3,4]. In essence, co-design seeks to facilitate shared decision-making by involving people in the decisions regarding their care to improve the quality of life and health outcomes for its key stakeholders [5].

There is increasing interest to involve older people in the creation of healthcare services [6], given that healthy ageing is now a global priority whereby people are living longer and are more likely to develop chronic or multiple health conditions [7]. More specifically, there is a current trend in creating digital health technologies in light of its ability for health and social care professionals to reach people in their home setting and support self-management and health literacy. A recent systematic review [8] highlighted how researchers are increasingly utilising co-design methods to create such technologies to support older people’s well-being and independence. The review concluded that supportive technology for ageing should continue to involve older people and that it is crucial to address age-related barriers unique to older people, particularly in relation to trust/relationship and knowledge building, when undertaking future co-design research.

### Overview of The ValueCare Project

The ValueCare Project [9] is a European research project, funded by Horizon2020, which seeks to co-design and deliver integrated care to older people living with chronic health conditions to improve their quality of life, as well as their care providers, and support the sustainability of healthcare systems in Europe, including 17 Consortium partners and seven large-scale pilots. The vision of the project is to co-design value-based integrated care with key stakeholders (older people, family or informal caregivers and health and social care providers) which will be supported by digital health technology and tested in a multinational pilot. The ValueCare Project and its inclusive co-design approach align with several components of the nine pillars of integrated care [10], namely digital solutions, people as partners in care and shared values and vision.

In Ireland, the aim of the study is to create technology to support older people living with mild or moderate frailty in the community setting of Cork and Kerry. Digital health technology is intended to promote and support a healthy lifestyle and behavioural changes while connecting them to community-based health and social care providers. In this context, frailty refers to a distinct health condition related to the ageing process whereby multiple physiological systems gradually lose their intrinsic capacity, yet the effects can be delayed and reversed with timely and appropriate interventions such as supported self-management and care planning [11]. Recent figures in Ireland show that frailty affects 21.5% of people over the age of 65 in Ireland [12]. Frailty can result in a significant increase in the number of times an older person visited their general practitioner and significantly impacted the rates of unplanned hospital care in Ireland [13]. Additionally, people with frailty in Ireland are eight times more likely to need homecare services than individuals not experiencing a level of frailty. As such, frailty has a significant cost and impact on both older people in their need in accessing necessary care and the health service to provide the level of care to manage the condition.

The ValueCare Project pilot seeks to improve the frailty integrated care pathway using a co-designed digital health technology that is grounded in the experiences of individuals living with frailty and their care providers in Ireland.

### Rationale for co-designing with older people in a digital setting

While the intended research project co-design methodology and protocol followed the traditional iterative, in-person workshops to create the digital health technology, the Covid-19 pandemic meant that standard in-person procedures for data collection were suspended. This event compelled the research team to rapidly pivot their approach and utilize new methods to capture the lived experiences of older people from their home setting. Public health advice required physical social distancing, restricted social contacts and travel measures that limited Irish citizens from travelling 5km beyond their home setting or undertaking any intercounty journeys throughout 2020 and 2021 in Ireland. This was particularly challenging as the research team were based in Dublin and the target co-design participants and relevant clinical setting were in Cork and Kerry where distances range from 260–300 km to travel for the research team. More specifically, older people (i.e., individuals over the age of 70) or individuals with underlying health conditions were required to ‘cocoon’ meaning that these citizens were advised not to leave their homes and restrict all social contacts unless requiring essential care to mitigate the risk of mortality and safeguard against the virus [14]. While this cocooning period occurred from March to August 2020, recommendations continued for these groups to avoid social interaction and a series of societal lockdowns took place during the data collection period. Additionally, standard older person social groups and daycare centres were also suspended to limit the spread of the virus, which further restricted the participant recruitment for the co-design and accessing an existing group to take part.

While there is a lack of uniformity regarding the co-design process [8], there is also a lack of knowledge regarding conducting co-design in an online environment, which is particularly pertinent in collaborating with older people. Although evidence exists that older people have adequate or high levels of technological literacy [15], however, a recent statistic showed that 33% of people over the age of 65 in Ireland had never accessed the Internet [16]. Therefore, the research team were mindful that some of their target population may not have the required knowledge to participate, due to digital poverty, digital literacy, accessibility and/or confidence to engage [17,18].

Pivoting the co-design process to a digital setting meant that the research team could not use a number of the tools available during in-person co-design workshops such as traditional idea mapping or role-playing. As such, the research team could not simply directly transfer the intended methodology to a digital setting as it may not have been appropriate or effective in capturing older people’s lived experiences. Additionally, Sumner’s *et al.’s* [8] review found that engaging older people in co-design involves many potential barriers including relationship building, knowledge building and methods and skills in the co-design process. While this review focused on traditional in-person co-design, the research team had to prepare for overcoming these challenges, which may be amplified in an online setting, to collaborate with older people in this way.

Reflection is a crucial cognitive practice within the research field [19] to ensure rigour and transparency and Rosanna Hertz [20] further observed that researchers should not simply report research findings but question and explain how findings are obtained. Given that meaningful involvement with patients and the public is at the core of co-design in healthcare improvement, the current article contains practical steps and considerations when collaborating with older people within a digital setting.

## Methodology

### Study design

Within this project, co-design refers to the process of collaborating with older people to develop a new value-based integrated care model supported by a digital solution that promotes older person health and social goals, supports informal caregivers and improves healthcare professional working conditions in clinical practice. Co-design was chosen as an appropriate methodology to explore and identify the values and care needs of older people in order to develop an integrated care model. Acknowledging the iterative nature of co-design [4], two rounds of co-design were conducted: the first round was conducted to assess older people’s priorities and preferences regarding their care as they age, while the second round focused on discussing potential solutions to meet these needs, based on the data gathered from the first round. The first round of co-design was conducted between November 2020 to July 2021, while the second round of co-design was undertaken between June and November 2021. While there are no specific guidelines regarding the frequency of co-design activities [21], the research team determined that conducting two sessions would allow the appropriate space to discuss their care experiences and attitudes toward potential solutions. The decision was further informed by the project requirements and how gatekeepers also advised that it would be more effective for older people to have two shorter sessions than one longer session which may be burdensome. Ethical approval for the study was granted by University College Dublin Research Ethics Committee (Ref. LS-21-69-Darley).

### Co-design approach

A combination of focus groups and interviews were chosen for this component of the study to gather qualitative data. Focus groups and one-to-one interviews were conducted via teleconferencing software and telephone methods. While traditional co-design is typically conducted using in-person workshops, focus groups were chosen for the digital setting to capture lived experiences within a group setting. This approach was particularly pertinent in understanding the needs of older people and the condition of frailty given that a group dynamic can make visible how people articulate and justify their own ideas in relation to others [22]. Nevertheless, the research team also included the option of one-to-one interviews (via telephone) to be inclusive of individuals who would be unable to access the digital setting or did not feel comfortable discussing their experience in a group environment. Focus groups were conducted by both the lead researcher and older person researcher, while the one-to-one interviews were conducted by the lead researcher. Additionally, both forms of data collection aligned with the intended data analysis method of inductive thematic analysis.

Acknowledging the focus of the research on the care of older people, the research team included an older person representative who guided the development of co-design study materials and the data collection process. Both research team members were appointed following the grant approval. The researcher who identified as an older person applied for the position and held extensive knowledge of the healthcare system due to their previous career as a healthcare manager. Both researchers learned about the co-design process together once appointed and before conducting data collection. This also involved several discussions and reflections amongst the researchers on how best to engage and make participants comfortable. The lead researcher maintained a journal throughout the co-design process containing field notes with collaborators, gatekeepers and co-design participants, as well as personal reflections regarding the ongoing progress of the co-design process.

### Participants

In total, five focus groups and twelve one-to-one interviews with older people were conducted over the course of the two rounds of co-design. The inclusion criteria were older people (75+ years of age), living with mild to moderate frailty in their home setting in Cork or Kerry. Although frailty is a recognisable and common phenomenon in ageing, within an Irish context, frailty is not a medical diagnosis because it can have different drivers (and hence different underlying diagnoses) in different people. The gold standard for the assessment and management of frailty is the Comprehensive Geriatric Assessment (CGA), however not all people living with frailty will receive this assessment within their care to receive a diagnosis [23]. As such, participants did not require a clinical assessment or diagnosis to participate in the research. Participants were required to self-identify or be identified by their gatekeeper to be an individual with challenges in the activities of daily living as an indication of experiencing mild to moderate frailty. Prior knowledge or experience with digital communication mediums was not a requirement to participate.

#### Participant recruitment

As the research team could not meet or engage potential participants in person, it was essential that the aims and objectives of the study were explained clearly and what would be expected of older people if they decided to participate. It also involved ensuring that participants were equipped to use technology to access the online setting if they wished to use it.

To access participants, the research team recruited local gatekeepers from organisations that actively engage older people e.g., day-care centres, age advocacy groups and older person councils. The National Steering Group in Ireland which comprised organisation representatives who worked in the area of older person care and integrated care advised on potential effective gatekeepers. As the research team were keen to gain urban and rural perspectives to represent the region, they contacted local community initiatives for recruitment. All gatekeepers involved were not known to or had a prior relationship with the research team and, similarly, were unable to meet in person to discuss the project. Therefore, the research team needed to meet gatekeepers using teleconferencing to explain the study (particularly to explain the study requirements) so that these gatekeepers were mobilised with knowledge when approaching potential participants. This meant that the gatekeepers introduced the study, in broad terms, to older people who could then agree to be contacted by a member of the research team (including name and organisation of the researcher) to discuss it in more detail. Gatekeepers were also pivotal in explaining the needs of older people to the research team and sharing their expertise such as avoiding the medical term ‘frailty’ as it has negative associations and the need for short periods for interviews and focus group sessions.

A member of the research team contacted each potential participant by telephone to explain the study and allow them to ask any questions and discuss the consent process. This communication also enabled the researcher to build rapport with each participant on an individual basis, before taking part in the co-design data collection and gaining an initial understanding of their circumstances. This researcher conducted the one-to-one interviews and was one of the focus group facilitators which helped build rapport with participants and gain initial insight into their technological needs. However, to avoid potential bias, the researcher remained impartial and explicitly stated that there was no obligation to take part and, where relevant, their gatekeeper would not be informed whether they chose to participate or not. A follow-up invitation letter was sent either by post or email to the interested participants containing a short overview of the project and sample questions to indicate the focus of the research: “*As you grow older, what are the challenges you face in your day-to-day life*?”, “*What do you do to promote your well-being*?”. This letter was designed to orientate potential participants and highlight topics that they may be asked to speak about. Accompanying the Participant Information Leaflet and Consent form, the researcher sent an animated explainer video of the project (https://www.youtube.com/watch?v=iu2ll18CDIE), created by the project’s Communications partner which provided a narrative overview of the project aims. This short video portrayed an older person who received integrated care and how an app may help in this process. The aim was to highlight the aim of the research and highlight the value of their input into its design.

Once they verbally agreed to participate, participants were required to provide written informed consent. As the research team could not gain consent in person, each participant was provided with the Consent Form and a stamped addressed envelope in which they could sign and return to the research team by post. For participants who provided email addresses and had personal access to a printer, they chose to print, sign and scan their Consent Form to the research team. Verbal consent was provided by each participant at the beginning of the co-design session. Participants were given the option to receive the full set of questions for the first focus group/interview ahead of time. This initiative was to familiarise participants with the aims of the co-design session and provide time for them to consider their answers in advance of the session. These questions included:

1. As you grow older, what are the challenges your face in your day-to-day life?
2. How would you like to be supported in addressing these challenges?
3. Do you think any of the challenges of growing older could be addressed by technology?
4. If you could design an app/technology, what would you like it to do for you?

As the aim of the research was to co-design digital health technology to support integrated care, it was important to understand older people’s existing knowledge of and engagement with technology. The research team developed a digital literacy survey that was specific to older people to assess their understanding of devices, apps and communication tools (Appendix A). The survey was created by the lead researcher in collaboration with a researcher of a fellow pilot site at Erasmus Medical Centre. Questions were adapted from other technology literacy surveys, however, were not specific to older people. The survey was reviewed by an age advocacy group to ensure comprehensibility and avoid bias. The survey was piloted with two older people before administration.

This short survey was completed with the researcher before their co-design session whereby they could indicate which technology, if any, they used, for what purpose, how often and whether they required help in using it. The survey contained a plain English explanation of what technology refers to and images of the specific devices (i.e., laptop, smartphone, apps) to help participants understand the questions. This survey also included demographic questions which were age-sensitive i.e., asking their age at the end of the survey rather than the beginning.

For participants who requested to take part using teleconferencing, it was essential for the research team to support them in setting up the software and training them in how to use it. This involved a short Microsoft Word document, including screengrabs and lay-person download and user instructions that avoided technological jargon, to guide the participant in using Zoom. The research team also conducted training sessions with each participant in which the team member telephoned the participant while using their computer or laptop to guide them through accessing the software. The training sessions with participants lasted between 30–50 minutes. In some cases, the research team also liaised with the participants’ family members, with the consent of the older person, to help set up the software and provide help on the day of the session. Some family members advised the researcher on the language to use when explaining technology to their relative and how to best guide them in the session.

### Data collection

Following the engagement and education of older people to participate, further steps and considerations were necessary for the conduct of the data collection within the digital setting. The focus group and interview schedule were structured so that their priorities and preferences regarding their health and social care were discussed before proceeding with how a technological solution could potentially meet their values. In both the focus group and interview sessions, the research team suggested that participants may wish to use a pen and paper to write notes or thoughts down during the session and it was explained that they could take as much time as possible to consider their answers. The research team conducted the telephone interviews to ensure that the views of older people who did not own or use technology were not excluded from the co-design process and the design of the digital health technology. These interviews lasted approximately one hour and followed the same questions as the focus group. Though focus groups were the preferred method as the data collection method to gather and explore the social construction of their experience and examine similar or contrasting ideas, the interviews also provided rich information regarding their personal experience of integrated care services and how they believed they could be improved.

Before each group or interview via teleconferencing, a member of the research team commenced the Zoom call 20 minutes before its intended commencement to allow participants time to access and resolve any technical issues, if they arose. The researcher also gave participants a direct phone number in case they needed contact outside of the platform. Focus groups were facilitated by a male and a female facilitator (who identified as an older person) and held health psychology and nursing backgrounds respectively. The researchers’ professional backgrounds were disclosed to participants to enable them to understand their facilitators’ perspectives and that they were able to discuss their health challenges if they chose to. Focus groups in which participants were not known to each other and joined separately contained small samples, i.e., between three and four participants, following the advice of local gatekeepers, to increase the sense of safety and allow rapport amongst participants. However, in focus groups that were conducted during the second round of co-design, participants were accessed through daycare centres in which the research team were able to hold up to eight people with the assistance of a healthcare staff member who set up the teleconferencing and moved the camera when needed as a participant spoke.

Ground rules were established before commencing each session when all participants arrived, including turning the camera on, where possible, speaking one at a time, and their ability to pause or leave if they so wished. To protect each individual regarding their identity and data (as detailed in the Information Leaflet and Consent Form), the researchers explained that their participation or their potential withdrawal would not be confirmed or discussed with the gatekeeper who identified them. The lead researcher explained to participants that all data would be anonymised during the analysis process and there will be no link between their answers and personal information. In this way, it would not be possible for any person to identify their responses. In the instance of focus groups, participants were required to not discuss the content of the session afterwards or details of fellow attendees with others.

While icebreakers were planned for the focus group to build rapport and a sense of teamwork amongst participants (e.g., identifying common traits, favourite pastimes), these were not necessary as participants discussed and bonded over their experience of the pandemic and restrictions in their local area. Non-verbal cues were observed when conducting the online focus groups, however, these were not formally used in the analysis.

### Data analysis

Focus group and interview recordings were transcribed verbatim. All data were anonymized and each participant was assigned a code to conduct the analysis. Inductive thematic analysis was performed by the research team using the data transcripts [24]. This inductive method was chosen for its lack of assumptions regarding the patterns of meaning contained within the data [24] and is a method commonly employed in co-design research [25,26,27]. Analysis was conducted in parallel with data collection to ensure that any issues raised are explored in co-design sessions in the development of the solution.

## Discussion

Digital focus groups are valued for conducting qualitative data collection in instances when in-person gatherings are not feasible [28] which was the context of the research project’s co-design process, undertaken during the Covid-19 pandemic. Acknowledging that the Covid-19 pandemic meant that older people could not access standard care services or engage in society that would typically support their well-being, made it more pressing to reach them to discuss how integrated care digital health may best support them. Therefore, while it was necessary to adapt the medium in which the co-design sessions were held, using teleconferencing and telephone methods, the research team also were required to adapt the co-design inquiry and identify alternative ways of building relationships, connection and trust with older people. This was particularly important given that co-design has been critiqued for being tokenistic i.e., for the idea that this process is carried out to endorse pre-existing strategies and designs rather than being open to redesigning health systems based on the experiences of service users [29,30] and how some older people can experience challenges in terms of digital technology, especially considering that an age-based digital divide continues to exist in the context of Ireland [31].

Preparation was central to conducting co-design in a digital setting. Key features of conducting focus groups with older people include a relaxing atmosphere and that it is conducted within a safe environment with no distractions [32] which was more complex in a digital setting, as the research team had less control of the physical environment in which participants were joining from. The research team were mindful that in most cases older people would not have the opportunity to meet the researcher or their fellow participants in person before or during the focus group. This meant that the research team were required to develop rapport and trust outside of the session before conducting data collection.

Communicating and engaging with older people to explain the project and facilitate familiarisation with the co-design aim before the session helped enable the conversation and meaningfully involve participants in the research. As such, time and dedication from researchers are essential to be invested to support older people to use digital platforms to participate in the co-design process and ensure they are comfortable doing so. Moreover, researchers should schedule and commit to educating and resourcing their participants with the required technology before conducting data collection in a digital setting. Current findings echo Arthanat and colleagues’ [33] argument that mutual trust, patience and simplicity are central to teaching technological platforms to older people. This process enables researchers to gain an awareness of their technological abilities, regarding devices and software, and understand how to best support them.

The research team also included one-to-one interviews as they did not want to exclude older people who did not previously own or engage with technology. As co-design is traditionally conducted in a collaborative team setting, one concern held by the research team was that including one-to-one interviews would inhibit the collective and collaborative method. However, the team used an approach that meant *bringing forward* the understandings of previous interviews and focus groups, anonymously, so that interviewees had an insight into what their peers discussed previously and prompted discussion. This approach suggests that patient experience using co-design may not need to be exclusively collected within a group setting. Researchers can adopt a cyclical approach in which they ask the same set of questions decided as a research team but then share with participants the experiences of others, without identifying them, so that they feel they have a stimulus of a wider group to feel engaged with.

A key part of the co-design process was having an older person representative as a member of the research team. This meant that team members could collaborate on study materials and ensure they were age-friendly. This team member facilitated the focus groups in which they openly identified as an older person to participants during the session and was able to ask and relate questions to ideas that were meaningful to them if a concept was not understood. Additionally, the research project consortium group included a representative from a non-profit organisation that advocates for older person rights.

Nevertheless, one challenge of adopting teleconferencing and telephone interviews to conduct co-design is that it was not possible to visually present and discuss all content with those collaborating using the telephone. The research team was required to consider how to communicate information clearly to participants when they did not have a visual stimulus via telephone and explain the functionality of digital technology to individuals who had no prior experience with it. This meant avoiding all technological jargon, asking participants to visually imagine how the technology could work and how they would like to be supported. In instances when a participant could not visualise the technology, the researcher asked them more generally about how they prefer information to be presented and what content they would find helpful.

The digital literacy survey, conducted before the co-design sessions, was instrumental in understanding each participant’s baseline knowledge of technology which subsequently helped frame the questions during the data collection. However, while in-person physical cues and body language may be inhibited using digital mediums, some participants said that it was a positive outlet to talk to someone about the experience of growing older and the challenges they face in this way. Anonymity may have been helpful for older people as they could speak openly about their experience, yet others raised queries about whether their information would be shared with their healthcare providers, despite their confidentiality being stated in the Information Leaflet and Consent Form. It must be noted that support services contact details were not provided in the information leaflet. However, two participants reported significant loneliness and isolation as a result of the Covid-19 restrictions during the data collection. The researcher advised them to contact their appointed general practitioner if they felt they needed support.

Given that the research was conducted during the Covid-19 pandemic, the research team could not negate the context in which the research was conducted. As such, the discussion focused on their experience of being socially isolated and while that may have been important to them, the research team were required to find a balance between acknowledging and listening to their circumstances and gathering data to answer the research question. From an ethical perspective, it was important for the research team to recommend participants to visit their GP or access local support services if they expressed low mood or social isolation. Thus, older people’s values may be found through the discussion of their context and life circumstances that may not be directly related to the co-design topic or explicitly aware of themselves.

Although utilising digital communication methods to conduct co-design research has several considerations, the current methodological approach highlights how they can be useful tools to capture older people’s lived experience. Co-design in an online setting enables researchers to reach older people’s and bring them together that may otherwise not have been able to participate due to travel distance, health status or personal circumstances. For instance, focus groups featured participants from urban and rural areas in Cork and Kerry who may not have been able to meet in person due to geographical location. When organizing focus groups or interviews with older people, consideration must also be given to their lifestyle, appointments and care they receive. It was important for the research team to choose a time for data collection that suited all participants, as they did not want to infringe on their care routine and commitments.

Older people may be supported in this way by having training sessions with researchers either in small groups or individually in which they can practice during the preparatory stage. However, it may be important to use this tool as optional rather than required during a co-design session as older people may prefer to explain their answers rather than write them down. Therefore, a blended approach for using online interactive tools for idea mapping and other co-design tools may be preferable for future co-design activities.

Reflecting on how to refine the methodology for future co-design projects with older people, the research team would create an additional educational resource of instructional videos that participants could watch following the live demonstration of the teleconferencing platform with the research team to ensure they felt comfortable before taking part in the co-design sessions. Additionally, we would include an evaluation step for participants to rate how equipped they felt to join the session and if there are any additional considerations the research team could include for future co-design sessions. However, this would need to be short to avoid burdening participants in the research process.

## Conclusion

There is increasing interest to involve older people in the creation of integrated care services given that healthy ageing is now a global priority. Patient and public involvement research seeks to include the voices and values of people who the intended healthcare solutions and services are aimed at. A synergy exists between co-design or participatory research and integrated care for its ability to explore people’s values and experiences to inform the design and delivery of integrated care. Person-centred care is a fundamental pillar of existing models of integrated care [34,35] and people being involved as partners in care is one of the nine pillars of integrated care identified by the International Foundation of Integrated Care [10]. Acknowledging the complexity inherent in the delivery of integrated care [36], co-design seeks to collaboratively establish the lived experiences and their related complexities of participants and develop solutions rooted in these experiences to meet their unmet needs, which may be sustainable in real-world clinical settings [37,38].

Despite the lack of literature on co-designing with older people in a digital setting and the considerations it presented, these were not insurmountable and were effectively addressed using our methodological approach. In essence, our steps include:

- Identify, inform and engage relevant gatekeepers to the intended participant population.
- Inform potential participants about the topic and what to expect when collaborating in a digital setting using written and visual materials, where appropriate.
- Build rapport with potential participants while maintaining good research practice to avoid bias or a sense of obligation to participate.
- Assess technological literacy levels and support requirements before conducting data collection.
- Allocate time and resources to provide support and training to access the digital setting.
- Reflect on the process of engaging participants in this setting and how it can be refined for future recruitment.

Our steps and considerations set out in this article to collaborate with older people using technology were based on the philosophy of meeting participants where they are, their digital literacy levels and preferences for participation, adapting the approach to empower them to participate. While the methods employed were not seamless, they did grant us the opportunity to capture older people’s experience in a fair, equitable and meaningful way. Our steps describe how a research team can develop and manage relationships with older people in an online setting and open a collaborative and communicative space to conduct co-design. The efforts to ensure that participants were educated, equipped and felt safe in a digital environment, as well as keeping them informed of the project’s progress, were key components of undertaking the research and achieving its aims. Our steps and reflections echo Howlett’s [39] sentiments on how conducting research in a digital environment have allowed for new ways of working with participants and gain a glimpse into their daily life from afar. More specifically, this methodology allowed the researcher team to gain an insight into older persons’ level of digital literacy to participate in the co-design process.

Within this study, the research team had several roles: answering the research question, building and fostering participant relationships, empowering adults to engage, educating participants about the topic and technology, facilitating collaborative data collection, providing technological support and ensuring participants were effectively involved and felt comfortable in the co-design process. This article is intended to be a guide, including practical tips and materials, for other researchers undertaking co-design processes using digital communication platforms which have been compellingly embraced since the Covid-19 pandemic. As this was a time when society recognized the value of connecting with others using technology, older people may have been more willing to participate as teleconferencing became a prevalent norm in communication. Even though the methodological approach concerned the creation of a digital health intervention to support integrated care, the co-design approach outlined could be adapted to wider integrated care research areas that involve engaging older people in a digital environment. Although this article focuses on the experience of digital co-design with participants in urban and rural areas in a large region of Ireland, this approach could be adopted to gather wider national and international older person views on any area of integrated care from several countries that would otherwise not be feasible.

## Additional File

The additional file for this article can be found as follows:

10.5334/ijic.6546.s1 Appendix A. Digital Literacy and Demographics Survey.
