# Supplementary material for: Conducting Co-Design with Older People in a Digital Setting: Methodological Reflections and Recommendations
Source: Int J Integr Care. 2022 Dec 9;22(4):18. doi: 10.5334/ijic.6546 (PMC9733123; doi:10.5334/ijic.6546)
Supplement: Appendix A. — Digital Literacy and Demographics Survey. [file ijic-22-4-6546-s1.pdf]

Digital Literacy and Demographics Survey

The purpose of this survey is to gain an understanding of your knowledge and usage of technology in your everyday life. Technology refers to devices such as computers, phones, tablets that people use in their everyday lives. We are interested in finding out whether you use technology and how you feel about it. Images of the technology we refer to are contained throughout. We also have included some demographic questions. The survey should take 5-10 minutes to complete.

Part 1: Technology Usage and Preferences

1. Do you own or have access to a computer or laptop?

☐ Yes
☐ No

Computer / Laptop Examples:
Error!

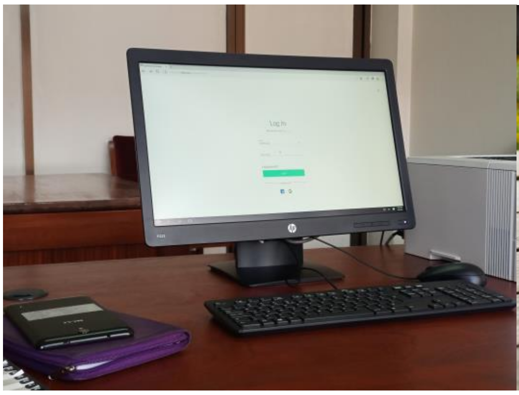

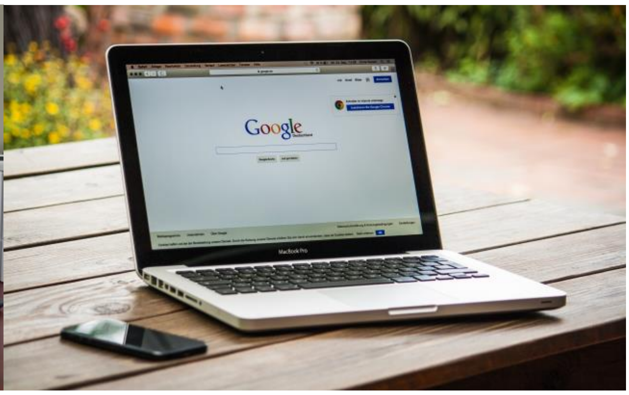

2. Do you use the Internet?

☐ Never
☐ Once or twice a month
☐ Once or twice a week
☐ Many times a week
☐ Everyday

3. **What do you use the Internet for?**

☐ Contacting family and friends (Phone calls, Text Messages, Whatsapp, Facetime, Zoom, Skype etc)
☐ Social Networking (Facebook, Twitter, Instagram etc)
☐ Reading the news
☐ Games, Puzzles, Crosswords
☐ Searching for health-related information
☐ Searching for other information
☐ Other (Please specify) ___________________________________

4. **(a) Do you own a mobile phone?**

☐ Yes
☐ No

(b) What type of phone?

☐ Mobile phone (i.e. A portable telephone with text messaging capability)
☐ Smartphone (i.e. A smartphone is a mobile device that combines telephone and computing functions)
☐ Senior mobile phone (i.e. a mobile phone with larger font and buttons designed for older people)

Mobile Phone

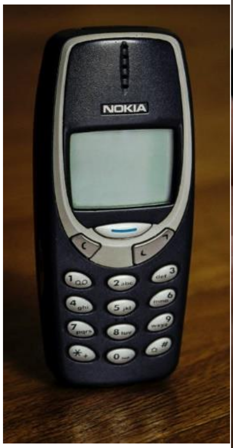

Smartphone

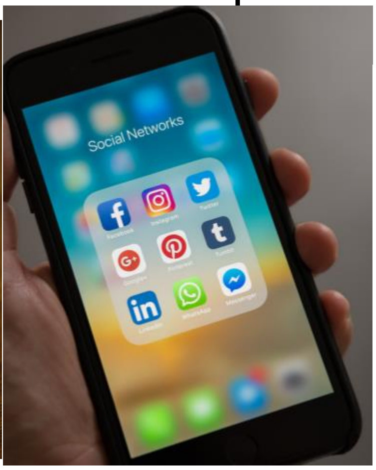

Senior-specific phone

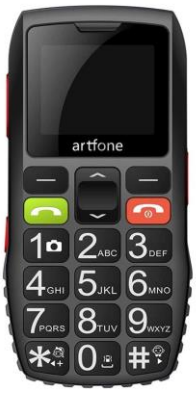

(c) If you do own a <u>smartphone</u>, how often do you use it?

☐ Never

☐ Once or twice a month

☐ Once or twice a week

☐ Many times a week

☐ Everyday

5. Do you own a tablet? (i.e. A portable touchscreen computer e.g. iPad)

☐ Yes

☐ No

Tablet Examples:

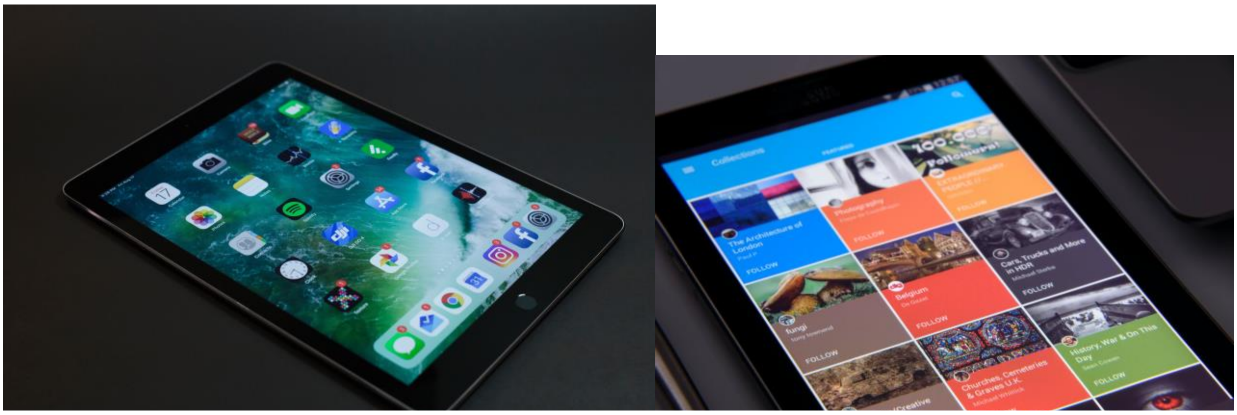

6. (a) Do you use apps? (i.e. Apps are applications that can be used on smartphones or tablets)

☐ Never

☐ Once or twice a month

☐ Once or twice a week

☐ Many times a week

☐ Everyday

Examples of Apps:

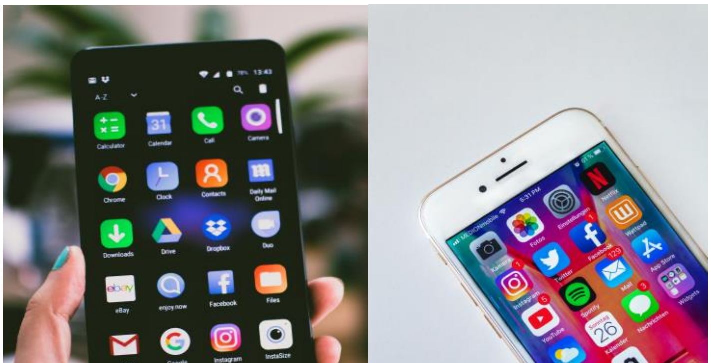

(b) If so, which categories of apps do you use?

☐ Communication (Whatsapp, Messenger, Zoom, Skype, Telegram)

☐ Social Media (Facebook, Twitter, Instagram)

☐ News

☐ Entertainment (Netflix, Spotify, Apple Music, Amazon Prime)

☐ Utility (Reminders, Calculators, Weather, Flashlight)

☐ Games / Puzzles

☐ Health and Fitness (MyFitnessPal, Headspace, FitBit, SleepScore)

☐ Other (Please specify)

____

____

____

____

7. **Do you ever require assistance from a family member or friend when using technology?**

☐ Never
☐ Rarely
☐ Sometimes
☐ Regularly
☐ All the time

8. **(a) Have you ever used/been prescribed any kind of technology as part of your healthcare?**

☐ Yes
☐ No

(b) If your answer is <u>NO</u>, would you like to use technology as part of your healthcare?

☐ Very Interested

☐ Somewhat Interested

☐ Neutral

☐ Not Very Interested

☐ Not At All Interested

(c) If your answer is YES, could you please mark which ones:

☐ Blood Pressure Monitor
☐ Blood Sugar Monitor
☐ Home Monitor or Sensors
☐ Falls Detector
☐ Activity tracker
☐ Personal alarms
☐ Medication reminders
☐ Online-based appointment booking system
☐ Virtual consultations with a health or social professional
☐ Online support forum
☐ Physiotherapy app
☐ Other ____

(d) Are any of these technologies standard practice as part of your healthcare? i.e. it is compulsory to use this technology as part of your care

☐ Blood Pressure Monitor
☐ Blood Sugar Monitor
☐ Home Monitor or Sensors
☐ Falls Detector
☐ Activity tracker
☐ Personal alarms
☐ Medication reminders
☐ Online-based appointment booking system
☐ Virtual consultations with a health or social professional
☐ Online support forum

☐ Physiotherapy app
☐ Other ____

Part 2. Demographics

1. Can you please state your age:

☐ 60-64
☐ 65-69
☐ 70-74
☐ 75-79
☐ 80-84
☐ 85 or older
☐ Rather not say

2. Can you please state gender:

☐ Male
☐ Female
☐ Rather not say

3. Can you please describe your living location:

☐ Urban/City (i.e. 50,000 people or more population)
☐ Town (i.e. Approximately 5,000 people)
☐ Rural (i.e. Low population area)
☐ Rather not say

4. Can you tick which one best describes your educational background:

☐ No education
☐ Primary education
☐ Lower secondary education
☐ Upper secondary education
☐ Post-secondary non-tertiary education
☐ Short-cycle tertiary education
☐ Bachelor or equivalent
☐ Master or equivalent
☐ Doctoral or equivalent

☐ Rather not say
☐ Other (Please specify) ___________________________________

5. **Can you tick which one best describes your type of residence:**

☐ Independent
☐ Living with Family
☐ Living in a household shared with others
☐ Residential Care/Nursing home
☐ Rather not say
☐ Other ___________________________________

6. **When were you diagnosed or made aware of your condition (to be specified depending on pilot site)**?

☐ Within the last 3 months
☐ Within the past year
☐ 1-2 years since diagnosis
☐ 3-5 years since diagnosis
☐ 5+ years since diagnosis

7. **Who is your main caregiver?**

☐ Not applicable
☐ One of your children
☐ Friend
☐ Neighbour
☐ Other (Please specify) ___________________________________
☐ Rather not say
